# The Study on Public-Interest Short Message Service (SMS) in China during the COVID-19 Pandemic: Mobile User Survey and Content Analysis

**DOI:** 10.3390/ijerph18157915

**Published:** 2021-07-26

**Authors:** Zhiyuan Yu, Yanghongyun Liu, Yongan Yu, Hongju Han, Yalin Li

**Affiliations:** 1 School of Journalism and Communication, Shandong University, Jinan 250100, China; yuzhiyuan@sdu.edu.cn (Z.Y.); liuyhy@mail.sdu.edu.cn (Y.L.); hj_han@mail.sdu.edu.cn (H.H.); liyalin@mail.sdu.edu.cn (Y.L.); 2 School of Physical Education, Shandong University, Jinan 250061, China

**Keywords:** COVID-19, public-interest short message service, epidemic prevention and healthcare, demographic difference, content analysis

## Abstract

The outbreak of coronavirus disease 2019 (COVID-19) has greatly threatened the global health system and triggered the public health emergency. In order to manage the COVID-19 pandemic, healthcare and prevention information have been delivered through omni-media channels (e.g., television, radio, social platform, etc.). As a traditional outlet, the short message service (SMS) can timely provide abundant anti-epidemic alerts to mobile users. In this paper, we aim to investigate mobile users’ attitudes toward COVID-19 public-interest SMS sent from government authorities and then explore the insight from messaging texts collected between January and April 2020 in China. In general, respondents show a positive attitude towards content and the necessity of public-interest SMS during the pandemic. However, we find that gender and age differences not only affect content evaluation, but also influence reading and forwarding behaviors. For the necessity of SMS, it shows significant difference between the 18–25-year-old and over 40-year-old group, with the middle and elder group showing serious attitudes and giving higher remarks than the youth due to the habits of media usage. However no significant difference is presented between females and males. In terms of content, the category of topics and releasing institutions are analyzed, respectively. Due to the centralized responses and coordination of prevention and control in China, the messages from COVID-19 disposal organizations (e.g., municipal steering group and provincial CDC) account for more than 70% among four cities.

## 1. Introduction

Since the 1990s, short message service (SMS) has gradually become popular and widely used from all works of life around the world due to its advantages of low costs, effectiveness, high arrival, and open rate. For individuals, SMS provide a flexible channel to chat with other people and institutions on a daily basis, such as sending text messages and querying bills. Moreover, a messaging text can be read and written conveniently via mobile phone, whenever and wherever, even if there is no internet access. For institutions and business companies, SMS can directly reach target users for distributing public-interest information (e.g., governmental affairs, health information, etc.) and advertising (e.g., commercial promotion). In order to dispense messages for a tremendous number of mobile users, telecom operators (e.g., Orange, Vodafone, Verizon wireless, and China Unicom) and business platforms (e.g., zipwhip, Tatango, and TextMagic) render help for both government and commercial organizations. In China, until December 2020, the accumulated number of short messages was up to 1779.57 billion [1]. The annual amount and its revenues in 2020 are increasing 18.1% and 2.4%, respectively [2]. In the U.S., 56% of surveyed companies make their schedules by SMS according to Zipwhip’s report [3]. We can see that SMS still has the most engagement for both individuals and organizations under the impact of social media.

### 1.1. Literature Review: SMS Application Areas

Many researchers have emphasized that SMS plays a vital role in fields of health communication, medicine treatment, momentous emergency events, etc. As an effective tool and intervention method, a series of studies have revealed the positive effects of sending health information by one-way or an interactive way for pre-exposure prevention [4]. SMS can help people manage their health [5,6] and prevent disease [7,8]. In particular, due to its cheaper price, it is feasible to conduct public health interventions in low- and middle-income countries [9]. Through SMS, the immunization coverage rate of the intervention group is significantly higher than that of the non-intervention group [10]. SMS can increase the virus re-detection rate and reduce the possibility of disease transmission [11]. For pregnant women, short messages are not only beneficial to the health of a newborn [6], but also maternal health and behaviors [5]. In addition, some unhealthy behaviors (e.g., smoking [12], sedentary [13], excessive calorie intake [14], etc.) can be rectified with the help of SMS reminders.

In terms of disease treatment, patients show high acceptance regarding SMS [15,16], so that it becomes the viable way for disease control and management [17,18,19,20]. In a health service platform, caring messages are continuously delivered to patients via SMS [21], e.g., medication reminders [16,22] and to perform healthy behaviors [23]. As a result, the compliance of patients with self-care can be further improved [24,25], which is specific in the attendance rate of outpatient appointments [26,27,28]. In a web-based disease management system, it is feasible to use interactive SMS diaries to collect patient’s disease data, i.e., patients send the messages of self-monitored physical conditions and receive disease management advice from doctors. Kwon et al. show that the effectiveness of this system is similar with face-to-face guidance and treatment [29].

For momentous emergency events and crises communication, government institutions usually distribute public-interest information via SMS, e.g., Amber Alert initially adopted in America [30]. Amber Alert, known as a warning system can quickly deliver information to surrounding people if cases of abduction or missing children are reported [31]. Afterwards, many countries, such as Canada, Malaysia, Russia, Netherlands, France, etc., have deployed similar systems. Based on the analysis, the Amber Alerts have a positive effect in solving child kidnapping issues [32,33,34,35] and 988 children have been successfully recovered up to May 2020 [30]. The “Crisis Text Line” provided by SMS is used in suicide clusters to prevent suicidal behaviors [36]. The caring and greeting messages were sent to soldiers, which could reduce the possibility of suicidal ideation and behavior [37]. Reese et al. found that SMS could help children to disclose maltreatment and seek support when abusive behaviors occur [38].

In term of public health emergency, Madagascar sentinel health centres detect incipient disease outbreaks through collecting daily syndromic cases by SMS to enhance disease surveillance capabilities [39]. The Ugandan Ministry of Health deployed a reporting system (RapidSMS) based on SMS in 2009. Once the platform received the reported message, medical staff would be noticed automatically [40]. Riha et al. applied interactive SMS to obtain social insights into Somalia after the 2017 cholera outbreak [41]. In order to control the spread of diseases in high risk areas, personalized contents were used to persuade people to change their travel behavior in space and time, which is viewed as a cost-effective intervention method [42].

After the outbreak of the COVID-19 pandemic, SMS provided by governments and operators has been used to fight against the epidemic alone with channels of radio, television, and social media. BT on behalf of the British government sent SMS reminders to approximately 43 million customers with clear advice to stay home [43]. Vodafone Spain enabled health professionals to disseminate important information through SMS [44]. In China, the central and local governments sent healthcare and prevention text messaging via operators of China Mobile/Unicom/Telecom. The SMS tracking platform was used by French physicians to monitor and advise COVID-19 patients after leaving emergency care [45].

### 1.2. Research Questions

Based on our knowledge, few researchers have focused on the study of public-interest SMS during the COVID-19 pandemic. In this paper, we aim to reveal mobile users’ attitudes toward anti-epidemic public-interest SMS by an online survey and then analyze the messaging texts to find insights. The valuable experiences (or lessons) are expected to draw from the case study in China, which can further improve the effects of SMS.

In order to conduct analysis and find insight, following the priori investigations of SMS as an intervention method in [15,16,18,23,26,46,47,48,49,50,51,52,53,54,55], we chose gender and age as influencing factors to further explore the attitudes’ differences towards COVID-19 public-interest SMS in research questions. Cho et al. adopted gender and age as the controlled factors and then indicated significant differences between females and males on attitudes towards SMS [46]. In Norway, there was also a significant gender difference in that women tended to be more frequent users of SMS than men [47]. Taylor et al. revealed that the ages of non-attended patients were younger than the attended-patients after sending SMS reminders for their next appointment, which showed a significant difference [55]. The research questions (RQ) are proposed as follows:

- RQ 1: What is the gender difference in evaluating the contents and necessity of SMS among male and female mobile users?
- RQ 2: What is the gender difference in reading and forwarding behaviors for SMS?
- RQ 3: What is the age difference effect in evaluating the contents and necessity of SMS among 18–25 and over 40 groups?
- RQ 4: What is the age difference in reading and forwarding behaviors for SMS?

## 2. Survey of COVID-19 Public-Interest SMS

The COVID-19 public-interest SMS refers to text messaging that contains topics of prevention and control regulations, self-health management and precaution knowledge, consumer prices, business guidance, fraud prevention tips, etc., which are sent from government authorities through operators (e.g., China Mobile/Unicom/Telecom) during the COVID-19 pandemic. As a direct channel to the public, public-interest SMS can provide useful and timely anti-epidemic official messages and allows mobile users to be informed with credible sources. For example, *Four “early” methods should be done to prevent and control the novel coronavirus pneumonia: Early discovery of symptoms relating to fever and dry cough, early get a diagnosis, early isolation if confirmed, and early treatment. Authoritative information can be obtained by subscribing to the Wechat account named “Health Hubei”* (Source: Hubei COVID-19 Prevention and Control Headquarter; date and time: 28 January 2020 13:54).

### 2.1. Data Collection and Measurement

We conduct an online survey from 1 May 2020 to 11 May 2020 via WJX (https://www.wjx.cn/) in China, with the related questions in the questionnaire are presented in Part S1 of Supplementary. After the period where the COVID-19 pandemic was regionally controlled, especial in the city of Wuhan, Hubei Province (Wuhan lifted a 76-day lockdown on 8 April and China announced that the COVID-19 control had obtained major strategic results for nation-wide and decisive results in Hubei and Wuhan on 29 April 2020). On the one hand, we shared the questionnaire link to social media moments (e.g., Wechat) to gather respondents. After finishing the survey, those participants also shared the questionnaire, in which the snowball sampling method was adopted. On the other hand, in order to extend the administrative regions of participants, the same questionnaire was distributed on survey platform. In this way, we have tried to increase the number of participants and collect samples representing the population from 34 provincial-level administrative regions of China. Specifically, we removed invalid questionnaires based on the following criteria: (1) With the foreign IP address and self-reported overseas location and (2) who had not received the COVID-19 SMS. A total of 1277 respondents completed the questionnaire and 1253 respondents were deemed as valid. The effective recovery rate of the questionnaire is 98.1%.

As a result, the questionnaires came from 31 provincial-level administrative regions of China, which include Shandong, Jiangsu, Hebei, Beijing, Henan, Hubei, Laoning, Shanghai, Zhejiang, Anhui, Sichuan, Fujian, Guangdong, Chongqing, Hunan, Shaanxi, Jilin, Shanxi, Tianjin, Heilongjiang, Yunnan, Xinjiang, Gansu, etc. The regional differences ensured the universality of the questionnaire results. The demographic characteristics are presented in Table 1. A total of 56.9% of valid respondents are female and 43.1% are male. In terms of age distribution, participants between 18–25 years old and 31–40 years old are in the majority, which account for 37.9% and 22.3%, respectively; 0.7% participants are under 18 years and 22.6% participants were above 40 years. For the occupation, 31.44% of the respondents are full-time students and 14.5% are from the education industry. According to self-reported geographic locations, we categorized those provinces into seven administrative regions, respectively.

In order to discriminate the effective participants, the respondent was asked to report a reading situation from seven items, i.e., “1 = almost carefully read each message”, “2 = carefully read selective contents based on topics or pandemic conditions”, “3 = almost skim-read each message”, “4 = selective skim-reading”, “5 = rarely read”, “6 = block (set blacklist) and never receive again”, and “7 = never read”. Therefore, we can prevent those who never browse or read COVID-19 messages for the next question.

Among the 1230 of 1253 valid respondents who used to read COVID-19 public-interest messaging, we measured the respondent’s attitudes toward the content, frequency, and necessity of COVID-19 messages, separately. The score of contents ranged from “1 = not helpful” to “10 = very helpful”. Meanwhile the respondents were asked to evaluate the frequency of COVID-19 public-interest SMS from “1 = lower frequency” to “10 = highly frequency”. Moreover, the indicator of necessity of COVID-19 SMS varied from “1 = extremely needed” to “5 = not at all” among 1221 respondents. In addition, the forwarding behavior also measured from “1 = never forward”, “2 = selective forward”, and “3 = forward each message”. After computing descriptive statistics, Student’s t-tests and $\chi^{2}$ tests are calculated to present the differences between genders and age groups. p<0.05 is considered statistically significant.

### 2.2. Results and Discussions

**Figure 1:**
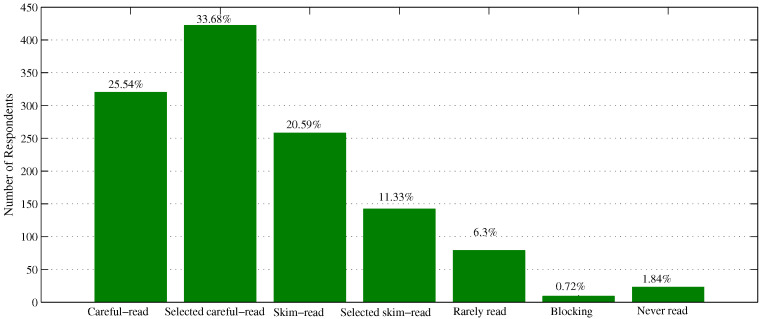
Reading situation.

Table 2 summarizes the basic statistics of the questionnaire. For the reading situation as shown in Figure 1, there is 59.22% of respondents who carefully read each or part of COVID-19 public-interest messages in the past few months. The other 31.92% of the overall sample, skim-read the received messages. We can see that more than 90% of respondents browsed COVID-19-related short messages via their own mobile phones at least once, which provides a solid foundation to pursue the following investigations.

For the content of public-interest SMS, it shows that the respondents gave higher marks (M = 7.65, SD = 1.869) and recognize that the received contents are helpful. As shown in Table 3, 62.28% of samples believe that SMS delivered sufficient information and offered timely reminders of pandemic prevention polices and guidelines. Only 3.41% of respondents thought that contents from SMS are useless, due to the fact that they have additional outlets (i.e., social media platform and instant messaging applications) to obtain similar messages.

In terms of SMS frequency, the score is relatively high (M = 7.2, SD = 1.774) and shows strong reminders have been provided by mobile operators for respondents, which can increase the read rate of certain important COVID-19 messages. For the necessity of SMS, 82.48% of samples hold views that existing public-interest SMS is quite necessary (36.28% and 50% respondents selected the item of very necessity and necessity, respectively). Depending on the operators’ vertical channel, SMS is not out of date and still exerts its functions to satisfy the high demands of mobile users.

After receiving a SMS, we also evaluate the forwarding behavior and found that 43.1% of respondents would like to share the short message (include “forward each message” and “selected forward”) with their family members and friends. In addition to the fact of usefulness and effectiveness, a SMS sent from the government sectors and a COVID-19 steering group are regarded as trustworthy sources with a high degree of credibility.

#### 2.2.1. Gender Difference

From the perspectives of content evaluation, the necessity of SMS and reading and forwarding situations, we further analyze the gender differences between males and females by conducting independent *t*-test and Chi-square test in SPSS 25.0, respectively.

Table 4 presents the gender difference to score contents of public-interest SMS. The analysis reveals a significant difference between females and males (t(1230)=2.009, p<0.05), who used to read COVID-19 short messages. The results show that the average score of males (M = 7.78, SD = 1.93) is higher than that of females (M = 7.56, SD = 1.817). We can see the gender variable does have an effect on content evaluation, which answers the RQ 1a.

Table 5 describes the gender differences in the necessity of public-interest SMS. This analysis fails to reveal a significant difference between females and males, (*t*(1221) = 0.872, *p* = 0.502) and suggests that the level of female’s views on the importance of the public-interest SMS (M = 1.78, SD = 0.696) is similar to that of males (M = 1.81, SD = 0.775). The gender difference does not influence the respondents’ cognition regarding the necessity of COVID-19 SMS, which answers RQ 1b. Combined with the scores of content evaluation, we can see that both the male and female respondents hold positive attitudes on COVID-19 public-interest SMS.

Table 6 represents the reading and forwarding situations regarding males and females. The chi-square test results show that there exists a significant difference between male and female. For the reading situation presented in Table S1, 62.79% and 28.91% of male respondents prefer to carefully read and skim-read (both including detailed and selective way) texts, respectively. Correspondingly, 56.62% and 34.08% of females selected the same items. In this way, the RQ 2a is answered. As reported in [56], males and females are informed of COVID-19 messages through different channels (e.g., television for men and websites of local medical institutions for women). Due to the habits of media usage, the read behaviors of men differ from that of women regarding COVID-19 public-interest SMS. In terms of forwarding behavior (includes forwarding one-by-one and selectively) as shown in Table S2, the willingness of male respondents (48.5%) is much higher than that of females (38.9%). The results suggest that men would like to share COVID-19 public-interest short messages than women, which answers RQ 2b.

#### 2.2.2. Age Difference

Considering the habits of media usage, the age differences is regarded as one of main factors to affect attitudes toward SMS. We investigated two independent groups, i.e., respondents 18–25 years old and over 40 years old respectively, and then explore their attitudes for COVID-19 SMS.

In Table 7, those two groups show the significant difference when evaluating the content (t(740)=5.973, p<0.001) and necessity (t(735)=−3.13, p<0.01) of public-interest SMS via *t*-test. The group consists of middle and elderly-aged respondents that give a higher evaluation on content (M = 8.09, SD = 2.223) and necessity (M = 1.71, SD = 0.73) of SMS than younger-aged groups, which answers RQ 3.

Table 8 presents the reading and forwarding behaviors with respect to age groups. The chi-square test and Fisher’s exact test results (*p* < 0.001) show that there is a significant difference between young and middle-above age groups, which answers RQ 4a. Respondents above 40 years showed serious attitudes toward received short messages. For the reading situation presented in Table S3, 40.2% of middle-aged and elderly people carefully read almost each COVID-19 message, which is greater than youth respondents between 18–25 years (13.2%). Moreover, the youth prefer to selectively read messages based on the trends of the pandemic and topics of content, which accounts for 34%. The reason is that the younger generation is apt to use social applications (e.g., Wechat, Sina Weibo, Tencent QQ) to acquire COVID-19-related information, and thus deem SMS as a supplement channel directly from operators and government authorities. Moreover, $\ddot{O}$cal et al. found that the stress and overall emotional reactions caused by COVID-19 increase with age [57]. The elder respondents intend to carefully read the text messaging for self-health management.

In terms of forwarding behavior, the analysis reveals a significant difference between those two age groups, p<0.001, which answers RQ 4b. The cross-tabulation is shown in Table S4. In the 18–25-year-old group, 73.1% of respondents would not forward a public-interest SMS and only 26% of respondents selected to share content. However, 56.1% of samples in the over 40-years age group would like to selectively forward the content. Compared with elders, the youth are more likely to receive the same content via social media and thus it is not probable to forward the homogenized short messages.

## 3. SMS Content Analysis

### 3.1. Data Collection and Category

We collected COVID-19 public-interest messaging texts during the period of January to April 2020 among four cities, i.e., Wuhan in Hubei province, Jinan, and Linyi both in the Shandong province, and Langfang in the Hebei province. Table 9 shows the statistic results of collected public-interest messages from delivery time, as well as total and unique numbers (removing duplication). After data cleaning, a total of 635 messages were gathered as research materials.

In order to reveal trends in the COVID-19 pandemic and public-interest SMS in the cities of Wuhan, Jinan, Linyi, and Langfang, we also summarized the total number of COVID-19 public-interest SMS sent from government authorities and new confirmed cases among four cities, location by location, during the 14 weeks as shown in Table S5 in Supplementary. For example, in January and Feburary of Wuhan, the number of COVID-19 public-interest text messaging dramatically grew as new confirmed cases increased. In particular, the number of messages in the second and third week was two and three times than that of the first week. Since March, as new confirmed cases went down, the frequency of SMS gradually returned to a normal level. A similar trend can also be found in Jinan, the capital of the Shandong province.

### 3.2. Content Discussion

According to the advice from the World Health Organization [58], the precautionary behaviors as well as public service events mentioned in SMS are classified into nine categories, i.e., “individual protection”, “make environment safer”, “self-health management”, “migration register”, “safety reminder”, “greetings, encouragement and mourning”, “public service”, “resumption & reopen”, and “daily necessities reminder”. Based on the keywords or description in SMS, each message is labeled at least with one tag. The topic statistics are presented in Table 10. We can see that the topics concentrate on individual protection, migration register, and public service. In addition, for particular regions, the topics of self-health management and making the environment safer account for above 10%. Detailed analysis with respect to (w.r.t.) the content and information sources of public-interest SMS can be summarized as follow.

During the period of the COVID-19 pandemic, the public should take care of themselves and ensure daily protection. Therefore, the texts of public-interest SMS offer concrete illustrations to alert mobile users to the propose of lowering infection risk and staying safer during the pandemic.

On a level of individual protection, reminders of wearing a mask, cleaning hands, and maintaining social distance rank as the top three in terms of frequency and almost appear in each message. This is due to such precautions being recognized as the efficient way to keep people safer. For dinners, the individual serving portion is encouraged to prevent cross-infection, e.g., serving spoons and chopsticks when they share dishes. The eaters are also urged to avoid a crowded sitting around the dining room and canteen. In addition, the messaging texts tell the public how to maintain social distancing through detailed and actionable suggestions. For example, it would be better to refrain from gathering and face-to-face contact, maintain at least 1-metre physical distance away from others, and avoid going to crowded places in light of reducing risks.

In self-health management, SMS provide illustrations on how to monitor body’s symptoms, enhance immunity, maintain one’s mental health, and prevent disease. Therein, “measuring body temperature”, “timely medical treatment”, and “appropriate exercise” are mentioned with a higher frequency in this category. The reminders of disease prevention include not only the symptoms of some chronic diseases (e.g., diabetes and hypertension) for the target group, but also the seasonal diseases (e.g., influenza and measles) for ordinary people. More importantly, many practical measures are advised to improve immunity, e.g., appropriate exercise, regular schedule, adequate sleep, and reasonable diet. Therein, according to BinDhim et al., who conducted a population health study in Saudi Arabia, the level of physical activity significant decreased [59], whereas sedentary behaviors tended to increase [60] during the COVID-19 pandemic. Ergo, there is an urge to encourage people to do proper indoor (or outdoor) exercises.

Making environment safe is vital to preventing the spread of COVID-19. Among all the contents belonging to this category, these reminders cover all types of places, e.g., home, community, urban space, transportation, school, and office. The top three topics in terms of frequency include indoor disinfection, opening the window, and indoor cleaning. In the early stage, the alters concentrate on home and community environment. For example, opening a window to allow fresh air flow inside, indoor cleaning and disinfection, airing the quilt and cloths frequently, waste sorting, and no spitting allowed in public area, etc. Due to this fact, most people stayed at home to learn or work remotely at that time. When COVID-19 had been controlled regionally in China, residents gradually returned to work and school. The number of alters w.r.t. the hygiene of office, school, and public transport environment tend to increase.

According to the collected short messages from cities of Wuhan, Jinan, and Linyi as shown in Table 10, the topics related to public service rank first and account for the highest proportion. Due to this category, it covers all kinds of services rendered in public interest. For example, tax policy, medical security, social insurance, transportation, combating illegal operations, and online and offline business handling are included in the category. Among them, there are three topics that are most often mentioned, i.e., transportation, medical security, and online and offline business handling. In order to satisfy the citizens’ needs, both central and local governments of China try their best to provide public services as usual. The concrete steps are taken to adapt the status of remote work, e.g., online application and approval, one-stop service with simplified procedures.

Specifically, we classify the resumption measures and polices as an independent category, i.e., work and school resumption. Most short messages involve protective measures, e.g., commuting protection and dos and don’ts for face-to-face learning. In Wuhan, there exists the reminders of traffic issues and livelihood services after recovering from lockdown. For enterprises, beside employee registration, some preferential treatments (or policies) are also announced to assist the return to work and production, for example tax reduction and reimbursement, and rental wavier. The name of polices and their query links are attached in texts for reference.

Daily necessity reminders can be regarded as a special subcategory of public service. This category mainly talks about commodity prices, goods delivery, and grain reserves derived from the department of development and reform (e.g., in Shandong) and COVID-19 heading group (e.g., in Hubei). The variation of necessities’ prices account for the majority. We can see that most of the texts contain the expressions of adequate supplying with a stable price. In virtue of those convincing information, stockpile purchasing behavior caused by rumor can be relieved. Meanwhile, the authority intends to guide rational consumption for necessary goods (e.g., food, mask, and sanitizer) during the COVID-19 pandemic.

The category of migration register includes entry declaration either from hometown or other places (even inside city) by scanning the QR code online and filling the format offline. It takes place in all crowded spots, e.g., community, shopping malls, transport vehicles, scenic zone, etc. For safety reminders, the messages either from a police office or COVID-19 leading groups promote mobile users to pay attention to fraud, use of disinfectant in a safe and effective way, and to avoid the leakage of personal information during the pandemic, which aims to help people get rid of being deceived and injured.

There are several topics related to greeting, encouragement, and mourning, such as greeting for a holiday, encouraging to boost morale against COVID-19, and mourning for compatriots who sacrificed due to COVID-19. Although this category does not account for a large proportion in overall SMS, it can improve the confidence of people who fight against COVID-19 and alleviates the anxiety of daily life.

In order to guarantee the effects of SMS, on the one hand, part of texts repeat the keywords many times to highlight their importance. For example, the repetition of the phrase “wear a mask” three times. On the other hand, duplicated short messages are frequently delivered to help the public focus on certain behaviors (e.g., measure temperature, entry declaration and migration registration, etc.) so that the public could follow the corresponding guidance and recommendations more likely, which will increase the level of personal protection and health management. In terms of language style, parallel construction with rhyme have been adopted, which is apt to read and impress deeply. The verbs of “advocate”, “suggestion”, “avoid”, and “enhance” are often used, which make expressions more clearly and concisely.

Public-interest SMS have the name of an authority to identify the source of content, which involves state, provincial, and municipal organizations in China. The top institutions in four cities are summarized in Table 11. We can see that the total number of messages from a majority sources account for more than 70%. In collected SMS datasets from Wuhan, Jinan, and Linyi, the municipal COVID-19 disposal group acts as the central organization to deliver public-interest messages. The reason is that after the outbreak of COVID-19 in China, governments at each level rapidly set up a joint mechanism to fight against the pandemic, which consists of all the functional departments. In Langfang, a majority of the content came from the Hebei provincial center of disease control and prevention (Hebei CDC), which accounts for above 90%. Several institutions were likely to jointly send public-interest content for a particular message, e.g., the Jinan Police Department, Transportation Bureau and Health Commission. The total number of joint issue ranks forth place in terms of frequency in Jinan.

## 4. Limitations

However, there exists some limitations in this survey. First, because of the maintenance of social distancing during the pandemic, most questionnaires are collected from the online survey platform or social media via the snowball sampling method. Although we screen each questionnaire to improve the quality of the questionnaire, the real behaviors of the respondents answering the questions could not be observed. Only people who can access the Internet are able to fill in the questionnaire. In future work, the combination of an online and offline survey should be adopted to obtain the questionnaires. Second, the majority age concentrate on 18–25- and 31–40-year-olds, the percentage of the elder age group (e.g., above 60 years old) is relatively lower than the others. A field investigation could be carried out in the community and old people homes to expand the age distribution. Third, differently to the previous SMS usage studies, we did not set up a control group to show the participants’ attitudes toward COVID-19 public-interest SMS during the pandemic period and other public-interest SMS after lifting lockdown.

## 5. Conclusions

In this paper, we have conducted an online survey to investigate Chinese mobile users’ attitudes toward the COVID-19 public-interest short message service (SMS). The demographic differences between gender and age have been revealed from aspects of content evaluation, necessity of SMS, and reading & forwarding behaviors. Overall, a large proportion of respondents have been revealed to hold positive and serious attitudes towards SMS. For SMS content collected from four cities, they render large scopes of COVID-19 information (e.g., prevention, healthcare and public service) directly from Chinese government authorities with higher credibility during the pandemic. The messages that come from top-releasing organizations (e.g., municipal COVID-19 disposal group and provincial CDC) account for more than 70% of the total number in each respective city.

In term of the implication of this study, we need to use the advantages of SMS as a supplement channel to distribute anti-pandemic messages for the public. Moreover, the messaging text should be carefully organized and well written to convey the main ideas for various and different mobile users, which aims to improve the effects of communication (e.g., persuade, alter, and encourage). In addition to the text form, depending on an updated SMS, named Rich Communication Services in 5G era, interactive messages with vivid pictures and audio-video could inform users by enhancing the quality of services. 

## Figures and Tables

**Table 1:** Demographic characteristics of the respondents.

Variables	Category	Percent	Variables	Category	Percent
Gender	Male	43.1	Region	North China	11.7
	Female	56.9		Northeast China	5.7
Age	<18 Years	0.7		East China	60.7
	18–25 Years	37.9		Central China	8.7
	26–30 Years	16.5		South China	3.0
	31–40 Years	22.3		Northwest China	3.8
	41–50 Years	10.4		Southwest China	6.4
	51–60 Years	10.2			
	>60 Years	2.0			

**Table 2:** Statistics of questionnaire item.

Question	Num.	Min.	Max.	Median	Mean	SD	Measurement Scale
Reading situation	1253	1	7	2	2.49	1.349	1–7
Content evaluation	1230	1	10	8	7.65	1.869	1–10
Frequency evaluation	1230	1	10	7	7.2	1.774	1–10
Necessity	1221	1	5	2	1.8	0.73	1–5
Forwarding situation	1221	1	3	1	1.45	0.531	1–3

**Table 3:** Participants’ comments on public-interest SMS.

	Num.	% of Respondents
Substantial content, timely reminder, helpful	766	62.28%
High credibility	791	64.31%
Homogeneous information learned from others	428	34.8%
Sending duplicated messages with redundant	512	41.63 %
Useless but it doesn’t matter	42	3.41%
No comment	24	1.95%
Other	15	1.22%

**Table 4:** Content evaluation of public-interest SMS between males and females.

Item	Groups	Number	Min	Max	Mean ± SD	Measure Scale	Sig. (Two-Tailed)
Content evaluation	Male	530	1	10	7.78 ± 1.930	1–10	0.045
	Female	700	1	10	7.56 ± 1.817	1–10	

**Table 5:** The necessity of public-interest SMS between males and females.

Item	Groups	Number	Min	Max	Mean ± SD	Measure Scale	Sig. (Two-Tailed)
Necessity of existence	Male	525	1	5	1.81 ± 0.775	1–5	0.502
	Female	696	1	5	1.78 ± 0.696	1–5	

**Table 6:** Reading and forwarding situations between males and females.

Item	Groups	Num.	Sig. (Two-Tailed)
SMS reading situation	Male	543	0.003
	Female	710	
SMS forwarding situation	Male	525	0.000
	Female	696	

Reading: 1 cells (7.1%) have expected a count less than 5. The minimum expected count is 3.90. Forwarding: 0 cells (0.0%) have expected for a count of less than 5. The minimum expected count is 9.03.

**Table 7:** Content evaluation and necessity of public-interest SMS in different ages.

Item	Groups	Number	Min	Max	Mean ± SD	Measure Scale	Sig. (Two-Tailed)
Content evaluation	18–25	461	1	10	7.15 ± 1.786	1–10	0.000
	over 40	279	1	10	8.09 ± 2.223	1–10	
Necessity of existence	18–25	457	1	5	1.88 ± 0.738	1–5	0.002
	over 40	278	1	5	1.71 ± 0.730	1–5		

**Table 8:** Forwarding and reading of short messages between ages.

Item	Groups	Number	Sig. (Two-Tailed)
SMS reading	18–25	476	0.000
	Over 40	281	
SMS forwarding	18–25	457	0.000
	Over 40	278	

Reading: 2 cells (14.3%) have expected a count less than 5. The minimum expected count is 1.86. Forwarding: 2 cells (33.3%) have expected a count less than 5. The minimum expected count is 3.03. The Sig. result is from Fisher’s exact test.

**Table 9:** Statistics of collected short message datasets.

Region	Receiving Period	Total Num.	Unique Num.
Wuhan, Hubei	24 January to 24 April	115	113
Jinan, Shandong	26 January to 30 April	408	118
Linyi, Shandong	30 January to 5 April	65	32
Langfang, Hebei	28 January to 28 April	47	47

**Table 10:** Topic statistics.

	SMS Data	Wuhan, Hubei		Jinan, Shandong		Linyi, Shandong		Langfang, Hebei	
Category		Num.	%	Num.	%	Num.	%	Num.	%
Individual protection		21	18.26	96	23.53	25	38.46	28	59.57
Self-health management		9	7.83	133	32.60	3	4.62	24	51.06
Make environment safer		9	7.83	41	10.05	8	12.30	18	38.30
Public service		71	61.74	177	43.38	21	32.31	0	0.00
Resumption & reopen		12	10.43	55	13.48	2	3.08	8	17.02
Daily necessities reminder		5	4.35	29	7.11	2	3.08	0	0.00
Migration register		22	19.13	164	40.20	20	30.77	3	6.38
Safety reminder		3	2.61	18	4.41	2	3.08	2	4.26
Greet, encourage, & mourn		14	12.17	20	4.90	2	3.08	1	2.13

Note: % = Number of mention/total number of SMS in respective region.

**Table 11:** Major source of public-interest SMS.

City	Institution	Num.	%
Wuhan, Hubei	Wuhan COVID-19 Prevention and Control Headquarter	85	73.91
	Hubei COVID-19 Prevention and Control Headquarters	16	13.91
Jinan, Shandong	Jinan Leading Group for COVID-19 Pandemic Disposal	259	63.48
	CPC Jinan Committee Leading Group of COVID-19	18	4.41
	Shandong Development and Reform Commission	17	4.17
	Jinan Police Department, Transportation Bureau, Health Commission	12	2.94
Langfang, Hebei	Hebei CDC	43	91.49
Linyi, Shandong	Linyi COVID-19 Prevention and Control Headquarter	25	38.46
	CPC Linyi Committee COVID-19 Disposal Headquarter	22	33.85

## Data Availability

The public survey data related to this paper has been presented and the others are securely protected by the researchers; SMS text data sets (only as a Chinese version) from the cities of Wuhan, Jinan, Linyi, and Langfang are available on reasonable request from the first author.

## Supplementary Materials

The following are available online at https://www.mdpi.com/article/10.3390/ijerph18157915/s1, Part 1: The related questions in questionnaire; Part 2: Table S1: Gender * Reading Situation Crosstabulation, Table S2: Gender * Forwarding Situation Crosstabulation, Table S3: Age * Reading Situation Crosstabulation, Table S4: Age * Forwarding situation Crosstabulation; Part 3: Table S5: Number of public-interest SMS and new confirmed case in the cities of Wuhan, Jinan, Linyi, and Langfang.

## Abbreviations

The following abbreviations are used in this manuscript:

COVID-19	Coronavirus disease 2019
CDC	Center of disease control and prevention
SMS	Short message service
