# Supplementary material for: The Study on Public-Interest Short Message Service (SMS) in China during the COVID-19 Pandemic: Mobile User Survey and Content Analysis
Source: Int J Environ Res Public Health. 2021 Jul 26;18(15):7915. doi: 10.3390/ijerph18157915 (PMC8345619; doi:10.3390/ijerph18157915)
Supplement: Supplementary file 1 [file ijerph-18-07915-s001.zip › ijerph-1277944-supplementary.pdf]

International Journal of
Environmental Research and Public Health

MDPI

Article

The Study on Public-Interest Short Message Service (SMS) in China during the COVID-19 Pandemic: Mobile User Survey and Content Analysis

Zhiyuan Yu [1], Yanghongyun Liu [1], Yongan Yu [2,*], Hongju Han [1] and Yalin Li [1]

[1] School of Journalism and Communication, Shandong University, Jinan 250100, Shandong, China; yuzhiyuan@sdu.edu.cn (Z.Y.); liuyhy@mail.sdu.edu.cn (Y.L.); hj_han@mail.sdu.edu.cn (H.H.); liyalin@mail.sdu.edu.cn (Y-L.L.)
[2] School of Physical Education, Shandong University, Jinan 250061, Shandong, China.;
* Correspondence: yuyongan@sdu.edu.cn

check for updates

Citation: Yu, Z.; Liu, Y.; Yu, Y.; Han, H.; Li, Y. The Study on Public-Interest Short Message Service (SMS) in China during the COVID-19 Pandemic: Mobile User Survey and Content Analysis. *Int. J. Environ. Res. Public Health* **2021**, *18*, 7915. https://doi.org/10.3390/ijerph18157915

Academic Editor: William A. Toscano

Received: 12 June 2021
Accepted: 22 July 2021
Published: 26 July 2021

Publisher's Note: MDPI stays neutral with regard to jurisdictional claims in published maps and institutional affiliations.

Copyright: © 2021 by the authors. Licensee MDPI, Basel, Switzerland. This article is an open access article distributed under the terms and conditions of the Creative Commons Attribution (CC BY) license (https://creativecommons.org/licenses/by/4.0/).

1. The related questions from Questionnaire

1) Gender:
☐ Male ☐ Female

2) Age:
☐ Under18
☐ 18-25
☐ 26-30
☐ 31-40
☐ 41-50
☐ 51-60
☐ Over 60

3) Region: ________

4) Have you received COVID-19 public-interest SMS from government/operators ? (e.g., prevention and control regulations, precaution knowledge, consumer prices, business guidance, fraud prevention tips, etc.)
☐ Yes ☐ No

5) What is your read situation about COVID-19 Public-Interest SMS?
☐ Careful-read almost each message
☐ Careful-read the selected message (based on contents, epidemic situation, etc.)
☐ Skim-read almost each message
☐ Selected browse (based on contents,epidemic situation, etc.)
☐ Rarely read
☐ Blocking
☐ Never read

6) Would you like to forward the received COVID-19 public-interest text messaging?
☐ Don't forward
☐ Selective forwarding
☐ Forward all one by one

7) Do you think the existence of COVID-19 public-interest SMS is necessary?
☐ Strongly agree
☐ Agree
☐ Neither agree nor disagree
☐ Disagree
☐ Strongly disagree

8) What is your opinion on the content of COVID-19 public-interest SMS? (multiple choice)

□ Substantial content, timely reminder, helpful
□ High credibility
□ Homogeneous information learned from others
□ Sending duplicated messages with redundant
□ Useless but it doesn't matter
□ No comment
□ Other

9) The overall content evaluation for COVID-19 public-interest SMS.
Helpless 1 2 3 4 5 6 7 8 9 10 Helpful
10) Please evaluate the sending frequency of COVID-19 public-interest SMS.
Lower frequency 1 2 3 4 5 6 7 8 9 10 higher frequency
11) Why you didn't read COVID-19 public-interest SMS? (multiple choice)

□ Do not have the habit of reading text messaging
□ Not interested in the content
□ Have other convient channels to obtain information (e.g., WeChat, Weibo, etc.)
□ Other

2. Reading & Forwarding Situation

Table S1. Gender * Reading Situation Crosstabulation.

		Male	Female	Total
Careful-read almost each message	count	168	152	320
	% of Total	13.4%	12.1%	25.5%
Careful-read the selected message (based on contents, epidemic situation, etc.)	count	173	249	422
	% of Total	13.8%	19.9%	33.7%
Skim-read almost each message	count	103	155	258
	% of Total	8.2%	12.4%	20.6%
Selected browse (based on contents, epidemic situation, etc.)	count	54	88	142
	% of Total	4.3%	7.0%	11.3%
Rarely read	count	27	52	79
	% of Total	2.2%	4.2%	6.3%
Blocking	count	5	4	9
	% of Total	0.4%	0.3%	0.7%
Never read	count	13	10	23
	% of Total	1.0%	0.8%	1.8%
Total	count	543	710	1253
	% of Total	43.3%	56.7%	100.0%

Table S2. Gender * Forwarding Situation Crosstabulation

		Male	Female	Total
Don't forward	count	270	425	695
	% of Total	22.1%	34.8%	56.9%
Selected forward	count	239	266	505
	% of Total	19.6%	21.8%	41.4%
Forward all one by one	count	16	5	21
	% of Total	1.3%	0.4%	1.7%
Total	count	525	696	1221
	% of Total	43.0%	57.0%	100.0%

Table S3. Age * Reading Situation Crosstabulation.

		Over 40	18-25	Total
Careful-read almost each message	count	113	63	176
	% of Total	14.9%	8.3%	23.2%
Careful-read the selected message (based on contents, epidemic situation, etc.)	count	75	162	237
	% of Total	9.9%	21.4%	31.3%
Skim-read almost each message	count	50	119	169
	% of Total	6.6%	15.7%	22.3%
Skim-read the selected message (based on contents, epidemic situation, etc.)	count	34	63	97
	% of Total	4.5%	8.3%	12.8%
Rarely read	count	6	50	56
	% of Total	0.8%	6.6%	7.4%
Blocking	count	1	4	5
	% of Total	0.1%	0.5%	0.7%
Never read	count	2	15	17
	% of Total	0.3%	2.0%	2.2%
Total	count	281	476	757
	% of Total	37.1%	62.9%	100.0%

Table S4. Age * Forwarding situation Crosstabulation.

		Over 40	18-25	Total
Don’t forward	count	118	334	452
	% of Total	16.1%	45.4%	61.5%
Selected forward	count	156	119	275
	% of Total	21.2%	16.2%	37.4%
Forward all one by one	count	4	4	8
	% of Total	0.5%	0.5%	1.1%
Total	count	278	457	735
	% of Total	37.8%	62.2%	100.0%

3. The trend of COVID-19 pandemic and public-interest SMS in the cities of Wuhan, Jinan, Linyi and Langfang

Table S5. Number of public-interest SMS and new confirmed case.

Week	Region	Num. of SMS	New confirmed cases
1st Jan. 24–30	Wuhan,Hubei	5	2144
	Jinan,Shandong	8	16
	Langfang,Hebei	4	9
	Linyi,Shandong	1	18
2nd Jan.31–Feb.6	Wuhan,Hubei	11	8979
	Jinan,Shandong	49	23
	Langfang,Hebei	3	6
	Linyi,Shandong	6	15
3rd Feb.7–13	Wuhan,Hubei	11	25287
	Jinan,Shandong	40	8
	Langfang,Hebei	3	14
	Linyi,Shandong	5	12
4th Feb.14–20	Wuhan,Hubei	15	9781
	Jinan,Shandong	41	0
	Langfang,Hebei	3	1
	Linyi,Shandong	14	2
5th Feb.21–27	Wuhan,Hubei	9	2733
	Jinan,Shandong	32	0
	Langfang,Hebei	7	0
	Linyi,Shandong	9	0
6th Feb.28–Mar.5	Wuhan,Hubei	7	1660
	Jinan,Shandong	32	0
	Langfang,Hebei	5	0
	Linyi,Shandong	5	0
7th Mar.6–12	Wuhan,Hubei	6	194
	Jinan,Shandong	42	0
	Langfang,Hebei	5	0
	Linyi,Shandong	7	0
8th Mar.13–19	Wuhan,Hubei	7	14
	Jinan,Shandong	32	0
	Langfang,Hebei	0	0
	Linyi,Shandong	9	0
9th Mar.20–26	Wuhan,Hubei	8	1
	Jinan,Shandong	23	0
	Langfang,Hebei	4	0
	Linyi,Shandong	0	0
10th Mar.27–Apr.2	Wuhan,Hubei	6	0
	Jinan,Shandong	21	0
	Langfang,Hebei	3	0
	Linyi,Shandong	6	0
11th Apr. 3–9	Wuhan,Hubei	7	1
	Jinan,Shandong	27	0
	Langfang,Hebei	2	0
	Linyi,Shandong	3	0
12th Apr.10–16	Wuhan,Hubei	5	0
	Jinan,Shandong	27	0
	Langfang,Hebei	4	0
	Linyi,Shandong	0	0
13th Apr.17–23	Wuhan,Hubei	9	0
	Jinan,Shandong	22	0
	Langfang,Hebei	3	0
	Linyi,Shandong	0	0
14th Apr. 24–30	Wuhan,Hubei	9	0
	Jinan,Shandong	12	0
	Langfang,Hebei	1	0
	Linyi,Shandong	0	0

The new confirmed data is from http://wjw.hubei.gov.cn/bmdt/dtyw/, http://wsjkw.hebei.gov.cn/xgyqtb/index.jhtml, http://jnmhc.jinan.gov.cn/col/col50366/index.html, http://lycdc.linyi.cn/tzgg.htm.
